# Impact of a Series of Educational Talks Taught by Health Professionals to Promote Healthy Snack Choices among Children

**DOI:** 10.3390/children8030203

**Published:** 2021-03-08

**Authors:** Víctor Arufe Giráldez, Javier Puñal Abelenda, Rubén Navarro-Patón, Alberto Sanmiguel-Rodríguez

**Affiliations:** 1 Education Faculty, Elviña University Campus, University of A Coruña, 15008 A Coruña, Spain; v.arufe@udc.es; 2 CEIP Eduardo Pondal de Ponteceso, Eduardo Blanco Amor, 1, Ponteceso, 15110 A Coruña, Spain; javierpunal2@gmail.com; 3 Facultad de Formación del Profesorado, Universidade de Santiago de Compostela, 27001 Lugo, Spain; 4 Facultad de Lenguas y Educación, Universidad Camilo José Cela, 28692 Madrid, Spain; asrgz2014@gmail.com; 5 Facultad de Lenguas y Educación, Universidad Nebrija, 28015 Madrid, Spain

**Keywords:** health, lifestyle, snacks, children, obesity, overweight, nutrition

## Abstract

Background: One of the great challenges facing today’s society is the need to combat overweight and obesity in schoolchildren. This study aimed to analyze the impact of a cycle of didactic talks—given to families by a specialist in pediatrics, a specialist in nutrition and dietetics and a specialist in physical exercise—on childrens’ snack choices and nutrition quality. Methods: A longitudinal, quasi-experimental and quantitative investigation was designed, working with a total sample of 50 students divided into control and experimental groups. The nutritional quality of daily snacks was recorded during the month before and the month after the cycle of talks given by health experts. Results: An increase in the nutritional quality of the snacks was observed in the days after the talk—but, after a week, values returned to normal. Conclusions: The giving of educational talks to promote healthy habits may have a positive impact on the nutritional quality of school snacks in the days immediately following the talks. However, some forgetfulness was detected over time, which reduced the nutritional quality of the snacks once more. For future work, it is recommended that researchers measure the impact produced by giving regular talks.

## 1. Introduction

Two of the 17 goals for sustainable development (listed in the 2030 agenda established by the United Nations (UN) to guarantee a healthy life) are (1) to promote the well-being of all ages and (2) to achieve improved nutrition. The UN stated that the vast majority of deaths are related to poor diet [1]. In parallel, the World Health Organization (WHO), based on multiple studies published in recent years, warned of the high rates of overweight and obesity in youth populations, accompanied by low levels of physical activity [2,3,4]. The institution warned of the need to promote a healthy lifestyle through different proposals that could lead to lifestyle changes—especially those influencing aspects of health, physical exercise and nutrition. In this sense, the WHO indicated that a healthy diet is one that avoids processed foods, energy-rich foods, fats (saturated and trans), free sugars and salt/sodium, and that seeks to increase the consumption of fruits, vegetables, legumes, nuts, unsaturated fats (e.g., fish, soy, olives) and other dietary fibers (e.g., whole grains).

The exact composition of a balanced diet varies depending on certain factors, including individual characteristics (e.g., age, gender, lifestyle, level of physical activity) or cultural context, among others.

Some authors have confirmed that motor competence and physical fitness in children correlate with to positive trajectories in physical activity, state of health and normal weight [5,6].

Combating childhood overweight and obesity has thus become one of the most important challenges of the 21st century. To meet this challenge, a key issue that must be addressed in order to improve the health status of children is the consolidation of a healthy diet. A correct diet—together with physical exercise—can contribute to decreases in the percentage of school-aged populations suffering from overweight and obesity. Currently, in the vast majority of countries, this percentage is around 20–30% [7]. Thus, it is stipulated that 2 or 3 out of every 10 schoolchildren are overweight or obese, with the consequent risk of contracting multiple pathologies associated with a sedentary lifestyle and poor diet. The increasing trend in overweight and obesity rates has already been noted by various authors in previously published studies [8,9], and although initially, the prevalence of overweight in children was associated with the most economically developed countries, a significant increase has also been observed in the greater part of the world [10].

High caloric intake, together with low levels of daily physical activity, causes the appearance of higher percentages of fat distributed in different areas of the body. It is likely that children with overweight/obesity will maintain their lifestyle into adulthood; thus, they may develop noncommunicable diseases such as diabetes and cardiovascular disease [11]. However, even in the short term, they can suffer from respiratory problems, hypertension, a higher risk of fractures (due to having a reduced capacity for balance) and problems related to mental health [12,13,14,15,16,17,18].

Some experts in the field of nutrition indicate that children’s snacks should improve in nutritional quality, avoiding high percentages of fat and carbohydrates [16]. Several investigations addressed the analysis of the most common low-cost and most-publicized snacks among schoolchildren, with the aim of studying the impact of potentially toxic metals (PTM) present in them on childrens’ health. These research papers associated a high weekly intake of PTM with carcinogenic risk, caused by certain metals and correlated with blood levels. The authors highlighted the need for initiatives aimed at improving nutritional quality and increasing awareness, as well as a PTM monitoring program in the low-cost snack market for young children [19].

In the infant and primary stages, the greatest responsibility for providing healthy snacks and meals to children falls on their parents, given the children’s lack of autonomy in food selection. The same does not happen in adolescence, where eating routines can be influenced by different structures and levels of control [20]. However, some researchers have studied the influence of the sources that provide nutritional information on the food choices (e.g., fruit or sweets) made by children. They confirmed that using experts to present nutrition information was a potentially successful strategy to raise awareness and present compelling arguments for children’s healthy food choices, in the face of less influence from celebrities and peers [21].

Some studies consider parents’ eating style to have an important influence on childrens’ dietary habits, with possible long-term effects [22]. A cohort study of 3626 families found that parental follow-up and family mealtime routines in early childhood provided a supportive eating environment that improved the overall quality of childrens’ diets. Thus, parents should choose healthy foods, not only for their children, but also to promote their own ability to self-regulate their food intake. A training program for parents on how to feed their young children should include strategies to encourage children’s preference for healthy foods and their acceptance of new foods, in addition to information related to the interpretation of labels [23,24]. Finally, it should be noted that a recent systematic review confirmed that—along with the genetic relationship—parents’ eating habits, lifestyle and dietary pattern influenced childhood overweight and obesity [25].

On this basis, the objective of this study was to analyze the impact produced by a cycle of didactic talks given to families by a specialist in pediatrics, a specialist in nutrition and dietetics and a specialist in physical exercise on children’s choice of snacks and their nutrition quality. It was investigated whether quality information from professionals in the Health and Sports Sciences to promote a healthy lifestyle in 6-year-old children influenced the decision-making of families when preparing and selecting snacks for their children, and if they were healthier, with lower percentages of carbohydrates and fats and a correct caloric intake.

## 2. Materials and Methods

### 2.1. Study Design and Participants

The study had a longitudinal, quantitative and a quasi-experimental research design. For the selection of the sample, the non-probabilistic convenience sampling technique was chosen due to its accessibility. The sample consisted of a total of 50 schoolchildren from the first year of Primary Education, with an average age of 6 years and assigned to a public educational center located in Spain. The respective parents of the schoolchildren in the sample also participated in the study and were the ones who received educational talks.

### 2.2. Process

For this study, two classrooms of 25 students from the 1st Year of Primary Education were selected. One of the classrooms made up the control group and another the experimental group. The educational center was selected through convenience sampling due to acceptance of the intervention by the authorities of the educational center. The educational center was a semi-urban center of public ownership.

In both groups, a daily record was made of the nutritional value of the snacks that the children brought to school over the course of a month. Each child was assigned a code, and the same researcher was always used to record these data so that the same criteria were used when analyzing the snacks. The nutritional value of the snacks was obtained through the official labels of the products, and in the case of not containing a label, it was extracted from the tables published in the Spanish Food Composition Database promoted by the Spanish Agency for Food Safety and Nutrition [26].

After the first month of registration, the families of the experimental group were invited to attend a cycle of three conferences given by professionals in the field of health (pediatrician), nutrition (Graduated in Nutrition and Human Dietetics) and physical exercise (Graduated in Sports Sciences). The talks were held in a classroom at the educational center, and all three sessions were held in a single afternoon in order to facilitate the attendance of families. During the talks, the three experts tried to promote among the families the acquisition of a healthy lifestyle in their children, providing current information on the rates of child overweight/obesity, sedentary lifestyle and the importance of taking care of diet and nutrition. The nutrition expert presented examples of different snacks considered healthy, as well as information on how to interpret nutritional labels. This information was based on healthy snack reports issued by international institutions such as the Healthy School Feeding Manual issued by the Food and Agriculture Organization of the United Nations (FAO).

The experts remained in the room 40 min after the talk and answered different questions raised by the children’s families. Since the talks were focused on an adult audience, no educational intervention was addressed in children.

The control group did not receive any talks. In both groups, the nutritional value of the children’s snacks was recorded for another month after the talks to analyze the possible nutritional changes in them.

### 2.3. Data Collection Instrument

For the collection of data related to the nutritional value of the snacks, an Excel sheet created ad hoc was used daily in order to record all the snacks that the schoolchildren consumed daily during the two months of the study. The data relating to the following snack variables were incorporated into this article: energy value expressed in kilocalories and percentages of fat (saturated, polysaturated, monounsaturated and trans), carbohydrates (with indication of sugars), proteins and salt. Subsequently, and after debugging, the data were incorporated into the SPSS statistical program for their treatment and analysis of the results.

### 2.4. Ethical Aspects

The entire research protocol was sent to the Ethics Committee of the national EDUCA platform for review and comments that could improve the research process, and it was accepted by said institution with code 12019. In addition, throughout the study, the work complied with the ethical recommendations reflected in various official documents and treaties on ethics in educational research, thus guaranteeing the anonymity of the participants, respect for them, confidentiality in the data reflected in the snacks, compliance with professional deontology and other ethical considerations related to research in education [27,28]. The families signed an informed consent form through which they agreed to participate in the research and allowed the registration of the snacks for statistical purposes. To avoid any type of influence on the research, they were not provided information on the objectives of the research, indicating only that information on the snacks would be collected to treat statistically and perform a descriptive analysis of these.

### 2.5. Statistical Analysis

The data were analyzed through the IBM © SPSS statistical program (version 25.0). A descriptive analysis was first carried out with the percentages of the different nutrients of the snacks consumed by the schoolchildren, and later a statistical analysis was carried out in which the descriptive statistics and differences were calculated according to the group variable (control vs. experimental). The normality tests (Shapiro–Wilk) revealed a normal distribution, using parametric tests (t-Student) for the comparison between the mean energy values according to the group (control vs. experimental) and moment (before and after talks by the experts). The level of significance was set at *p* ≤ 0.05 for the different tests.

## 3. Results

Regarding the descriptive analysis of the children’s snacks, it should be noted that in the first month, the most consumed snack was commercial cookies with chocolate chips, present in 21% of schoolchildren. Sandwiches were the second most consumed snack with a percentage of 18%, followed by other foods as can be seen in Table 1.

Little variety was observed in the snacks, since some snacks were consumed in very high quantities, and with three of them they would have comprised more than 50% of the total snacks. Post-talk, a different distribution of the snacks was observed, with a lower percentage corresponding to the less healthy ones than in the pre-talk snacks. This indicates that the predominant foods in the first phase lost prominence in this second, and in turn, there was greater variety in the foods consumed by the students. Among the foods considered “other” in the month after the talk, multiple types of fruit and other snacks considered healthy were recorded.

The means and standard deviations of the mean energy values were analyzed before the intervention of each of the groups (control and experimental), as well as the normality analysis of the data. The results of the Shapiro–Wilk test indicated that the data presented a normal distribution for energy values (*p* = 0.811), carbohydrate intake (*p* = 0.988), fat intake (*p* = 0.461), protein intake (*p* = 0.059), saturated fat (*p* = 0.732) and salt (*p* = 0.127), but not for sugar intake (*p* < 0.001).

The analysis carried out indicated that the groups were not homogeneous with respect to the variables analyzed before starting the intervention with health talks, as there were differences in pre-TEV (Total Energy Value) (t 48 = 3.213; *p* = 0.002); in Carbohydrates pre (t 48 = 2.255; *p* = 0.029); in Fats pre (t 48 = 4.155; *p* < 0.001) and in Salt pre (t 48 = 2.863; *p* = 0.007) (Table 2), these differences disappeared when applying the training program on healthy snacks in all variables comparing each other the experimental group and the control group.

If we compare the differences in each of the nutrients (i.e., TEV; Cbh; Saturated Fats; Fats; Sugar; Salt and Prot) before and after the process between the control and experimental group, it is observed that the students in the experimental group decreased their TEV (M = −18.41; SE = 18.26) versus the control group, in which it increased (M = 32.35; SE = 14.33, t (48) = 2.191, *p* = 0.033, r = 0.30). No statistically significant differences were found in the differences (diff) of the other nutrients before and after the intervention, although the experimental group (EG) experienced a decrease in the intake of each of them, while in the control group (CG) there was an increase (i.e., Cbr (difEG = −1.75; DifCG = 5.59, t (48) = 1.286, *p* = 0.205, r = 0.18); Fats (difEG = −3.08; DifCG = 0.51, t (48) = 1.923, *p* = 0.06, r = 0.26); Saturated Fats (difEG = −0.03; DifCG = 0.78, t (48) = 1.042, *p* = 0.303, r = 0.14)]. On the other hand, a decrease in intake was observed in both groups in the Prot (difEG = −4.47; DifCG = −1.39, t (48) = 1.569, *p* = 0.123, r = 0.22), in Salt (difEG = −0.10; DifCG = −0.05, t (48) = 1.923, *p* = 0.06, r = 0.26) and in Sugar (difEG = −21.24; DifCG = −2.77, t (48) = 1.195, *p* = 0.238, r = 0.17).

If the evolution of the control group is compared during the duration of the process, it is observed that there was a significant increase in TEV (t (24) = 2.265, *p* = 0.033, r = 0.42). There was also an increase in Cbh (t (24) = 1.438, *p* = 0.163, r = 0.28), in Saturated Fats (t (24) = 1.357, *p* = 0.187, r = 0.26) and in Fats (t (24) = 0.423, *p* = 0.676, r = 0.08). Intake in Prot (t (24) = −0.942, *p* = 0.355, r = 0.18), in Sugar (t (24) = −1.154, *p* = 0.260, r = 0.23) and in Salt (t (24) = −0.710, *p* = 0.485, r = 0.14).

If the evolution of the experimental group before and after the process is compared, it is observed that there was a statistically significant decrease in the intake of Prot (t (24) = −3.472, *p* = 0.002, r = 0.58) and in the Fats (t (24) = 2.182, *p* = 0.039, r = 0.40). there has also been a decrease, but not significantly, in TEV (t (24) = −1.008, *p* = 0.323, r = 0.20), in Cbr (t (24) = −0.419, *p* = 0.679, r = 0.08), in Saturated Fats (t (24) = −0.066, *p* = 0.948, r = 0.01), in Sugar (t (24) = −1.391, *p* = 0.177, r = 0.27) and in Salt (t (24) = 1.320, *p* = 0.199, r = 26).

**Figure 1:**
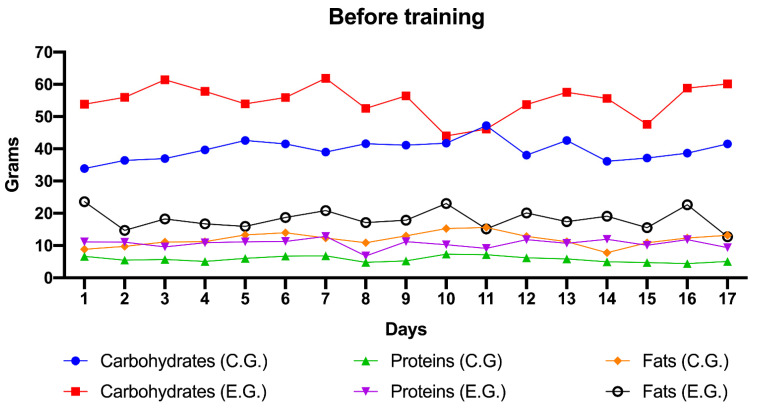
Values expressed in grams of carbohydrates, proteins and fats, of the control and experimental group’s snacks before training (daily data from day 1 to day 17). Note. C. G.: Control Group; E.G.: Experimental Group.

**Figure 2:**
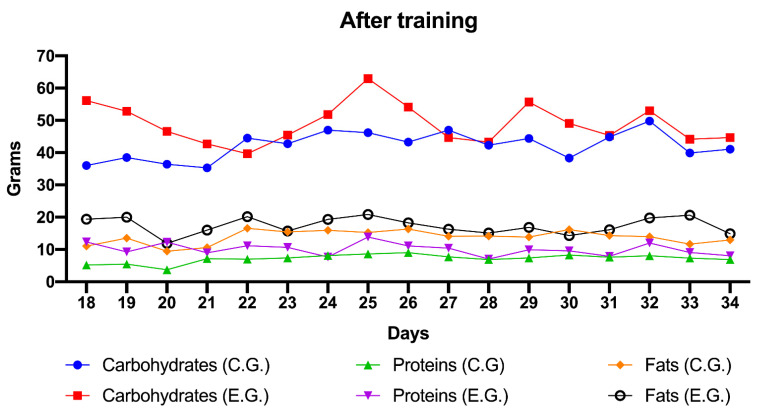
Values expressed in grams of carbohydrates, proteins and fats, of the control and experimental group’s snacks after training the experimental group (daily data from day 18 to day 37). Note. C. G.: Control Group; E.G.: Experimental Group.

In the following figures (Figure 1 and Figure 2) we can see the daily evolution of the control and experimental group regarding the consumption of carbohydrates, fats and proteins.

Before training, the experimental group had a higher intake of proteins, carbohydrates and fats than the control group, as can be seen in Figure 1.

Globally, if we compare the intake of each of the nutrients (i.e., carbohydrates, fats and proteins) in the control group and the experimental group before training, it is observed that the intake of all the nutrients is higher in the experimental group, with statistically significant differences in carbohydrates (*p* = 0.001) and fats (*p* < 0.001) but not in proteins (*p* = 0.059).

After the educational talk on healthy eating, the differences between the experimental group and the control group were equal (Figure 2).

Overall, if we compare the intake of each of the nutrients (i.e., carbohydrates, fats and proteins) in the control group and the experimental group after training, it is observed that the initial differences decreased, the only significant differences being in protein intake (*p* = 0.016), but not in carbohydrate intake (*p* = 0.081) or fat intake (*p* = 0.191), which tend to equalize.

## 4. Discussion

The concern with measuring the impact of educational talks to improve children’s lifestyles in relation to the nutritional value of snacks has not been widely studied by researchers, and few studies have been found that refer to this type of study. A longitudinal investigation carried out in a sample of 107 children applied an educational intervention given by a professional in the health field, confirming that after said intervention, the students in the experimental group obtained more knowledge related to diet and healthy lifestyle than the students in the control group, with this difference being statistically significant [29].

However, there are several studies that use other types of interventions aimed at children or families with children to promote the consumption of healthy foods. Thus, in this work [30] carried out in three rural counties of the United States, the impact of an educational macro-campaign to promote the consumption of healthy beverages and to raise awareness about the possible health dangers of consuming sugary beverages was evaluated. The authors found that there were no differences in terms of beliefs about harmfulness or consumption, and the doctors participating in the study affirmed that the intervention increased awareness, but it was insufficient to prompt action. They concluded that the public educational campaign on the harmfulness of sugary drinks and the provision of healthier options in some vending machines had a successful impact on the availability of soft drinks but did not reduce their consumption. These results are in line with this article’s work, where a certain influence is confirmed in the first days, especially in carbohydrates, which fell to their minimum value on the fifth day after a health talk, and fat percentage, with a less notable decrease in the first three days, but the educational intervention did not have enough impact to consolidate a healthy habit in the children’s families. This lack of adherence or commitment of parents to the selection of healthy snacks for children may also be due, among other factors, to incidental or intentional forgetfulness [31].

Regarding the nutritional quality of children’s snacks, in an investigation [32] with a sample of 212 children between 7 and 9 years old, it was found that when children consumed snacks after lunch and dinner based on fruits instead of sweet or salty snacks, a moderating effect of the absence of hunger is caused, thus obtaining a lower caloric intake and increasing the intake of healthy food. In another study [33], the effects of a school-based dietary intervention program to increase fruit and vegetable consumption among fourth-graders were evaluated, with researchers finding strong effects of the intervention on fruit and vegetable consumption, on macro and micronutrients and on psychosocial variables, while pointing out the need to carry out more intervention studies to test the effectiveness of the intervention when taught by classroom teachers.

In another quasi-experimental study [34] carried out in 126 mothers who had children between 1 and 5 years old and where an educational intervention was also applied based on four training sessions, a brochure, a short video and six written messages to analyze the frequency of consumption of the different types of food, a positive effect on the consumption of healthy snacks was shown. On the other hand, in the United Kingdom, a study was carried out [35] on a sample of 49 parents and adolescents aged 12 to 14 years, whose intervention to promote the consumption of fruits and vegetables was based on the distribution of an information bulletin on healthy habits sent by post. After 6 weeks, it was detected that the adolescents in the experimental group had significantly higher consumption of fruits (*p* < 0.001) and vegetables (*p* < 0.05) and higher preferences for vegetables (*p* < 0.001), compared to the control group, with similar results in parents. The authors concluded that interventions based on family newsletters promote fruit and vegetable consumption among adolescents. All these studies achieve results similar to those of this article’s research, observing that, to a lesser or greater extent, there is a change in food selection; however, the change in our study was not statistically significant in all nutrients. Another study indicates that parents sometimes do not know how to interpret what a healthy diet is [36]. They are aware of the importance of fruit and vegetables in their children’s diet and, in a way, they are motivated to give them a good diet. The authors of this study suggest that the information provided to families on nutritional habits is of vital importance to improve the nutritional status of children. Other researchers [37] analyzed the impact of two group training sessions and seven individual sessions on healthy nutrition in 73 healthy women. They confirmed that marital status, socioeconomic status, educational level, and household size did not appear to influence the dietary response, while women without children followed dietary advice more closely than women with children, so children also had some influence on the nutritional habits of their parents.

Finally, it is worth highlighting two studies that analyzed the type of snacks consumed by children and young people, through which an excess of caloric, fat and sugar consumption was confirmed. Thus, in a study carried out on a sample of close to 5000 adolescents [38], the prevalence of snack consumption throughout the day was analyzed, noting a high frequency of caloric, sugar and fat intake from unhealthy snacks, and recommending that the authors modify the type of snack to help adolescents to consume diets more in line with national recommendations. In another work [39] on 21,236 individuals between the ages of 2 and 18, it was concluded that unhealthy snacks are very common in society, recommending the establishment of programs that favor the choice of healthy snacks over high energy-dense snacks.

## 5. Conclusions

The objective of this research was to analyze the impact produced by giving a series of talks by various health and sports professionals to promote a healthy lifestyle in regard to children’s snacks. A positive impact was confirmed by an improvement in the nutritional quality in the days following the giving of the talks to the families. However, this impact fades after a few days and may be due to incidental or intentional forgetting. The days after the talks, the families were more aware of selecting healthy snacks, with a lower percentage of fat and carbohydrates, most likely because they still had the words of the different health and exercise professionals encouraging them to opt for a healthy lifestyle and good nutrition fresh in their memories, but after several days, the snacks returned to slightly lower fat, carbohydrate and calorie values than the days prior to the talk cycle. The sugar values decreased notably in the post-talk experimental group, but without being statistically significant. For most of the nutrient values, decreases are observed with respect to the month before the talk, but they do not become statistically significant in many cases.

## 6. Limitations and Future Research

It is proposed for future research work to analyze the impact of educational talks repeated over time, in order to consolidate the habit of selecting healthy snacks in the families of schoolchildren. Furthermore, more studies that address this same research protocol are required to confirm these results with other studies, since one of the limitations of this research was its sample size. Another suggestion for future work is to collect data related to children’s weight, body mass index as well as percentages of fat tissue. Future researchers who wish to tackle this line of research are also encouraged to combine different types of intervention registered in the scientific literature, thus creating a combined intervention of talks given by professionals, brochures and videos, and investigating their impact on the choice of the type of snack for children. It is necessary to continue to count on raising awareness about healthy habits in families and children to eradicate overweight and obesity and thus achieve the challenges and objectives established by the 2030 Agenda in relation to improving the health of the population.

## Figures and Tables

**Table 1:** Snacks most consumed by experimental group schoolchildren in the first month (pre-talk) and second month (post-talk).

Snack	Pre-Talk%	Post-Talk%	Snack	Pre-Talk%	Post-Talk%
Commercial cookies with chocolate chips	21	15	Processed orange juice	5	5
Cold cuts sandwich	18	10	Tangerine	3	5
Cocoa cream sandwich	12	10	Chocolate croissant	3	3
Snack breadsticks with sunflower seeds	11	10	Sobao bread or muffin	3	2
Banana	11	15	Corn cakes	2	4
Commercial strawberry petit-suisse	9	9	Others	2	12

**Table 2:** Descriptive data of the analyzed variables. Average, Standard Deviation, regarding pre- and post-training and Group (control vs. experimental).

		Control Group (C.G.)		Experimental Group (E.G.)		C.G. vs. E.G.
Variable	Time	M	SD	M	SD	*p*-Value (CI)
Total Energy Value (TEV) (kilocalories)	Pre-training	291.14	69.26	379.73	119.16	0.002 (33.15–144.00)
	Post-Training	323.60	32.45	361.31	89.32	0.056 (−0.51–75.00)
Carbohydrates (Cbh) (grams per product)	Pre-training	36.05	16.28	47.19	18.58	0.029 (1.20–21.07)
	Post-Training	41.64	9.62	45.43	14.99	0.292 (−3.37–10.95)
Protein (Prot) (grams per product)	Pre-training	9.18	6.89	13.22	7.84	0.059 (−0.16–8.24)
	Post-Training	7.79	1.41	8.75	4.33	0.296 (−0.86–2.79)
Fats (grams per product)	Pre-training	12.13	3.82	17.29	17.28	0.000 (2.66–7.64)
	Post-Training	12.65	5.23	14.21	7.69	0.407 (−2.18–5.29)
Saturated Fats (grams per product)	Pre-training	4.73	2.24	5.58	2.76	0.240 (−0.58–2.27)
	Post-Training	5.52	1.52	5.54	2.08	0.963 (−1.01–1.06)
Sugar (grams per product)	Pre-training	19.95	10.62	38.35	77.07	0.243 (−12.88–49.68)
	Post-Training	17.17	3.95	17.10	6.20	0.961 (−3.03–2.88)
Salt (grams per product)	Pre-training	0.56	0.177	0.83	0.43	0.007 (0.08–0.45)
	Post-Training	0.51	0.300	0.72	0.44	0.055 (−0.01–0.42)

M: Average. SD: Standard Deviation. Abbreviations: Total Energy Value (TEV); Carbohydrates (Cbh); Protein (Prot).

## Data Availability

Data sharing not applicable.
